# Genetic polymorphism of 29 STR loci in the Hunan Han population from China

**DOI:** 10.1080/20961790.2017.1306430

**Published:** 2017-05-08

**Authors:** Yanfang Liu, Ying Liu, Juanjuan Guo, Xiaoliang Fu, Zhihui Wang, Yujie Liu, Jifeng Cai, Lagabaiyila Zha

**Affiliations:** Department of Forensic Science, School of Basic Medical Sciences, Central South University, Changsha, China; Department of Oral and Maxillofacial Surgery, Xiangya Stomatological Hospital, Central South University, Changsha, China; Department of Forensic Science, School of Basic Medical Sciences, Central South University, Changsha, China; Department of Trade and Economic, School of Economy and Trade, Hunan University of Commerce, Changsha, China

## Dear Editor,

The Han population accounts for the majority of the Chinese population. Hunan is a large province located at 108°47′–114°15′ E and 30°08′–24°38′ N in Southern China, specifically in the area in the middle reaches of Yangtze River. The province has a total area of 218 400 km^2^ and a population of 72 million. The Han population of the province is approximately 59.15 million [1]. In this study, we evaluated the allele frequencies and forensic parameters of 29 short tandem repeat (STR) loci by using two commercial kits (Goldeneye™ 20A kit and Investigator HDplex kit). Bloodstain specimens were obtained from 501 unrelated healthy individuals (with informed consent) from the Han population of the Hunan Province, China. The Goldeneye™ 20A kit (PeopleSpot Inc., Beijing, China) includes the amelogenin locus and 19 autosomal loci (i.e. *D19S433*, *D5S818*, *D21S11*, *D18S51*, *D6S1043*, *D3S1358*, *D13S317*, *D7S820*, *D16S539*, *CSF1PO*, *Penta D*, *vWA*, *D8S1179*, *TPOX*, *Penta E*, *TH01*, *D12S391*, *D2S1338*, and *FGA*). The Investigator HDplex kit (Qiagen, Hilden, Germany) contains the amelogenin locus, one CODIS (*D18S51*), and 11 Non-CODIS loci (i.e. *D2S1360*, *D3S1744*, *D4S2366*, *D5S2500*, *SE33*, *D6S474*, *D7S1517*, *D8S1132*, *D10S2325*, *D12S391* and *D21S2055*). Both kits contain *D18S51* and *D12S391*, which can be used for sample concordance.

Genomic DNA was extracted from the bloodstain samples by using the Chelex-100 protocol according to the description by Walsh et al. [2]. DNA concentration was diluted to 1.0 ng/µL by adding high-purity water. Goldeneye™ 20A PCR kit was used following the manufacturer's guidelines [1]. Qiagen Investigator HDplex kit was used following the manufacturer's guidelines but reduce the volume to 10 µL [4]. PCR amplification was performed on a GeneAmp® PCR 9700 thermal cycler (Thermo Fisher Scientific, Waltham, MA, USA). Capillary electrophoresis was performed on an AB 3130XL Genetic Analyzer (Thermo Fisher Scientific). 9948a and nuclease-free water were amplified as positive and negative controls, respectively. DNA profiles were analysed with GeneMapper ID 3.2 (Thermo Fisher Scientific).

Genotype frequencies at each locus, power of discrimination (PD), power of exclusion (PE), probability of matching (PM), polymorphism information content (PIC), observed heterozygosity (Ho) and Hardy–Weinberg equilibrium (HWE) were evaluated with modified PowerStat (version 1.2) program [5]. Linkage disequilibrium (LD) analysis was performed using Genepop version 4.2 software package (http://genepop.curtin.edu.au). The allele frequency distributions among groups were compared using Arlequin version 3.5 software package [6]. Pairwise genetic distance among populations was calculated according to Nei's formula by using Phylogeny Inference Package (Phylip) version 3.69 [7]. Neighbour-Joining (NJ) phylogenetic tree was constructed and viewed with TreeView softwar [8].The current study was approved by the Third Xiangya Hospital of Central South University (approval code: 2016-S041).

The allele frequencies and population genetics parameters of the 29 STR loci are summarized in Table S1. We detected 403 alleles across the 29 STR loci, with the largest number of 50 variants at the *SE33* locus and the least number of 6 alleles at the *TPOX* locus. Genotyping results should be combined with the standard DNA allelic ladder to avoid genotyping errors because of the complex internal sequence structure of the *SE33* locus [9]. The *P*-value for Hardy–Weinberg testing did not significantly deviate from HWE after Bonferroni correction (*P* = 0.05/29 = 0.0017), only *D21S2055* (*P* = 0.000 2) and *D7S1517* (*P* = 0.0012) had minor departures from HWE because of errors in random sampling. Among the 29 STR loci, the *SE33* locus was the most informative marker, with a PD value of 0.9930 and a PIC value of 0.9500, whereas the *TPOX* locus presented the lowest forensic efficiency, with a PD value of 0.7590 and a PIC value of 0.5200. The Ho ranged from 0.5930 (*TPOX*) to 0.9240 (*Penta E*). The combined PM and combined PE for the 29 STR loci were 1.34 × 10^−36^ and 0.999 999 999 998, respectively. Pairwise LD tests demonstrated that only 18 pairs (Table S2) of the loci remained in LD for 406 pairwise comparisons after Bonferroni correction (*P* = 0.05/406 = 0.00012). These significant pairs in LD may be attributed to random sampling errors because the pairs are located on different autosomal chromosomes or chromosome arms.

Table S3 shows the locus-by-locus population differences (*P*-value) in the Investigator HDplex kit between the Hunan Han population and previously published data of other ethnic populations at the same loci. Evident differences (*P <* 0.05) were found between the Hunan Han and the Dutch [10], North Italian [11], Somali [12], Lithuanian (except *D3S1744*, *P =* 0.0723) [13] and Swedish (except *D3S1744*, *P =* 0.1267 and *D8S1132*, *P =* 0.2776) [12] populations. Significant differences were also observed from the Hunan Han population at *D2S1360*, *D3S1744*, *D10S2325*, *SE33* and *D12S391* with the Hebei Han [4]; at *D4S2366*, *D8S1132*, *SE33* and *D12S391* with the Sichuan Han [14]; and at *D3S1744*, *D10S2325*, *SE33* and *D12S391* with the Shanghai Han [15]. Table S4 shows the genetic distances between Hunan Han and other populations. Figure S1 presents the phylogenetic tree constructed by comparing the allelic frequencies of 11 STR loci in the Investigator HDplex kit and determines the genetic relationships of the Hunan Han population to the other populations. The minimum genetic distance and close genetic relationship were found between the Hunan Han and Shanghai Han (0.0059) populations. The largest distance was observed between the Hunan Han and Somali (0.1752) populations.

Our results for 19 STR loci in the Goldeneye™ 20A kit compared with other populations are also summarized. Table S5 shows that the distribution of alleles for the Hunan Han versus Suzhou Han [16], Henan Han [17], Yunnan Bai [18], Liaoning Manchu [19] and Philippine [20] populations indicated statistically significant differences at 8, 7, 14, 15 and 16 STR loci, respectively. No significant differences were found between the Hunan Han with the Guangdong Han [3] (except *D7S820*, *Penta E* and *TH01*), Huai'an Han [16] (except *Penta E*), Yangzhou Han [16] (except *D19S433*, *D3S1358* and *TH01*) and Taizhou Han [16] (except *D16S539*, *Penta D* and *TH01*) populations. Table S6 and Figure S2 present the genetic distances and phylogenetic tree between the Hunan Han and other populations, respectively. The minimum genetic distance and close genetic relationship were detected between the Hunan Han and Guangdong Han (0.0040) populations. The largest distance was found between the Hunan Han and Liaoning Manchu (0.0618) populations.

In summary, data suggested that the use of 29 STR loci could provide highly informative polymorphic data for forensic identification and establish a database for the Hunan Han group in China. The data also demonstrated that the distribution of allele frequencies varied in different areas or even in the same area for different subpopulations. Close genetic distance and genetic relationship were noted among regions separated by small distances because of their geographic proximity and high gene mobility.

This paper strictly follows all the guidelines for publication of population data as required by this target journal.

## Supplementary Material

1306430_suppl.zip

1306430_suppl.zip
