# Supplementary figures and images for: Genetic polymorphism of 29 STR loci in the Hunan Han population from China
Source: Forensic Sci Res. 2017 May 8;4(4):351–3. doi: 10.1080/20961790.2017.1306430 (PMC6968709; doi:10.1080/20961790.2017.1306430)

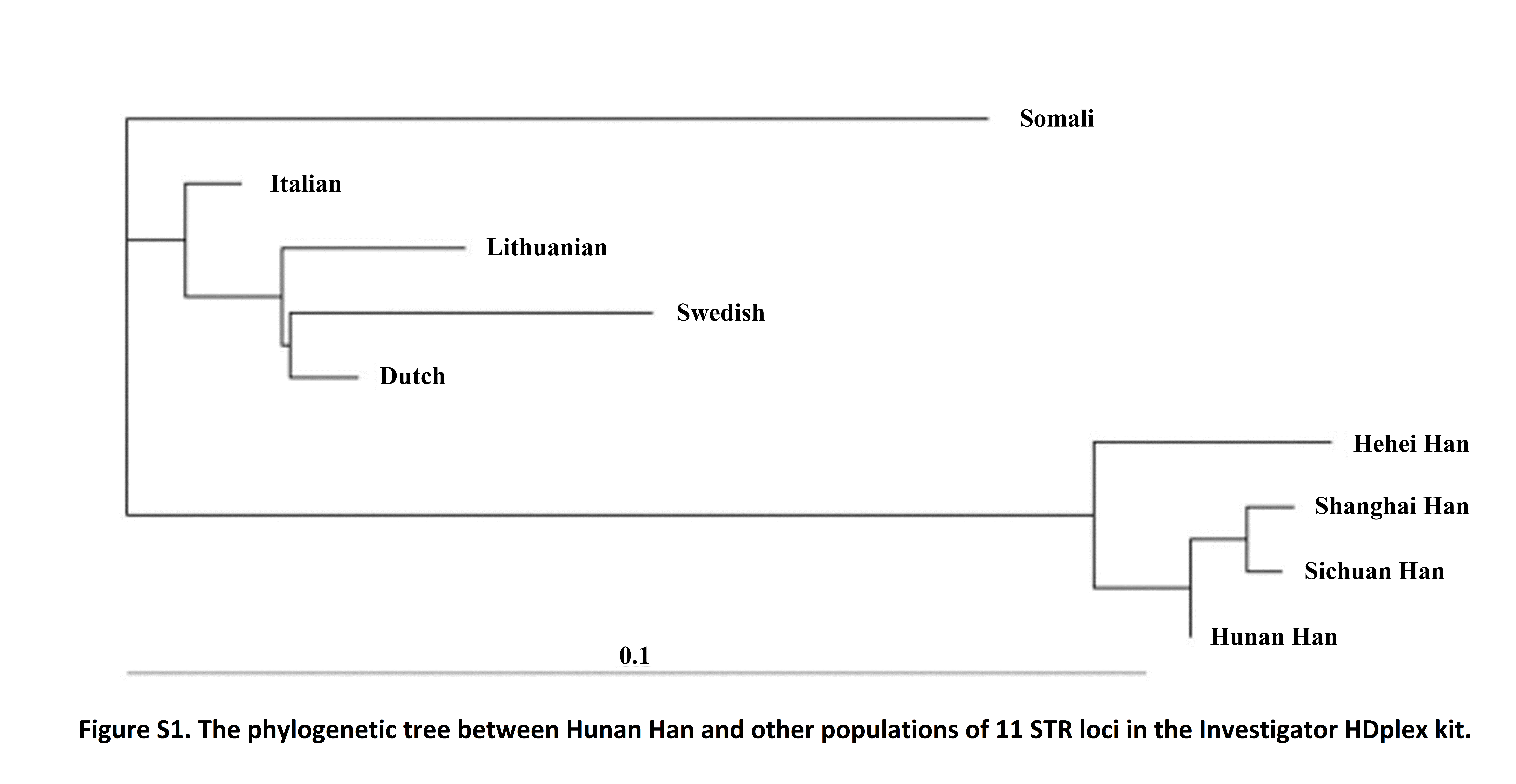

Supplement: 1306430_suppl.zip [file TFSR_A_1306430_SM2219.zip › 1306430_suppl/Figure S1 .tif]

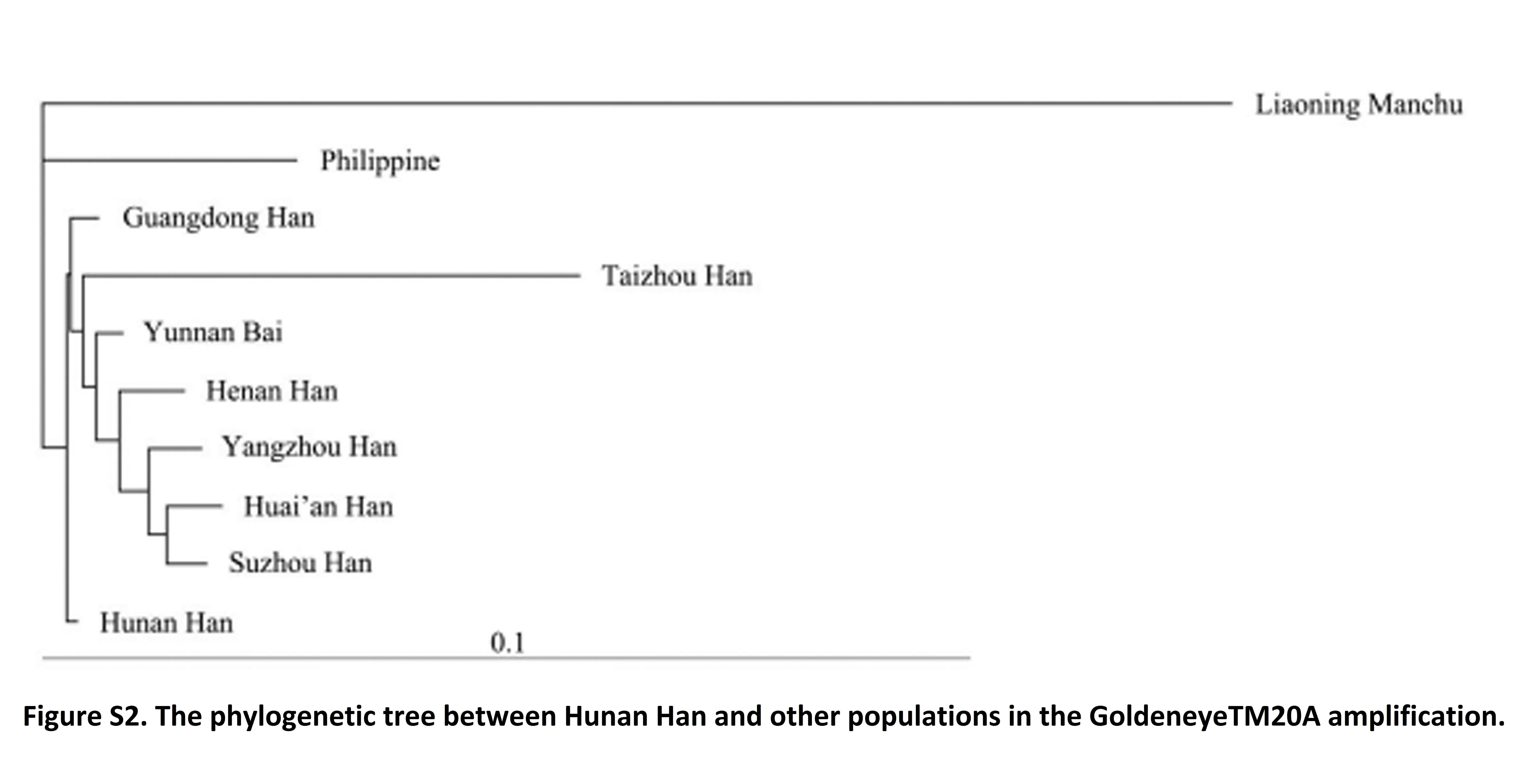

Supplement: 1306430_suppl.zip [file TFSR_A_1306430_SM2219.zip › 1306430_suppl/Figure S2.tif]

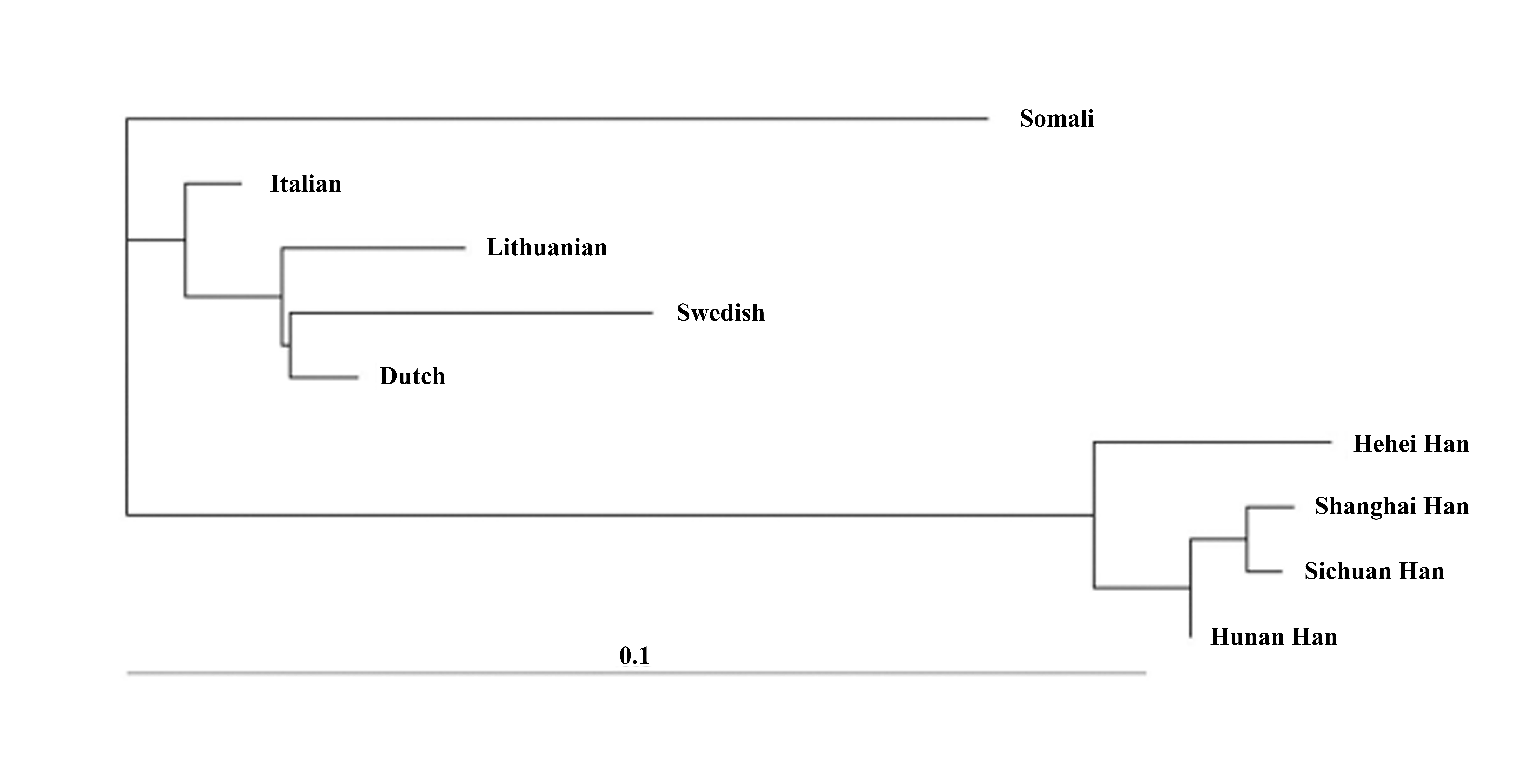

Supplement: 1306430_suppl.zip [file TFSR_A_1306430_SM5902.zip › 1306430_suppl/Figure S1 .tif]

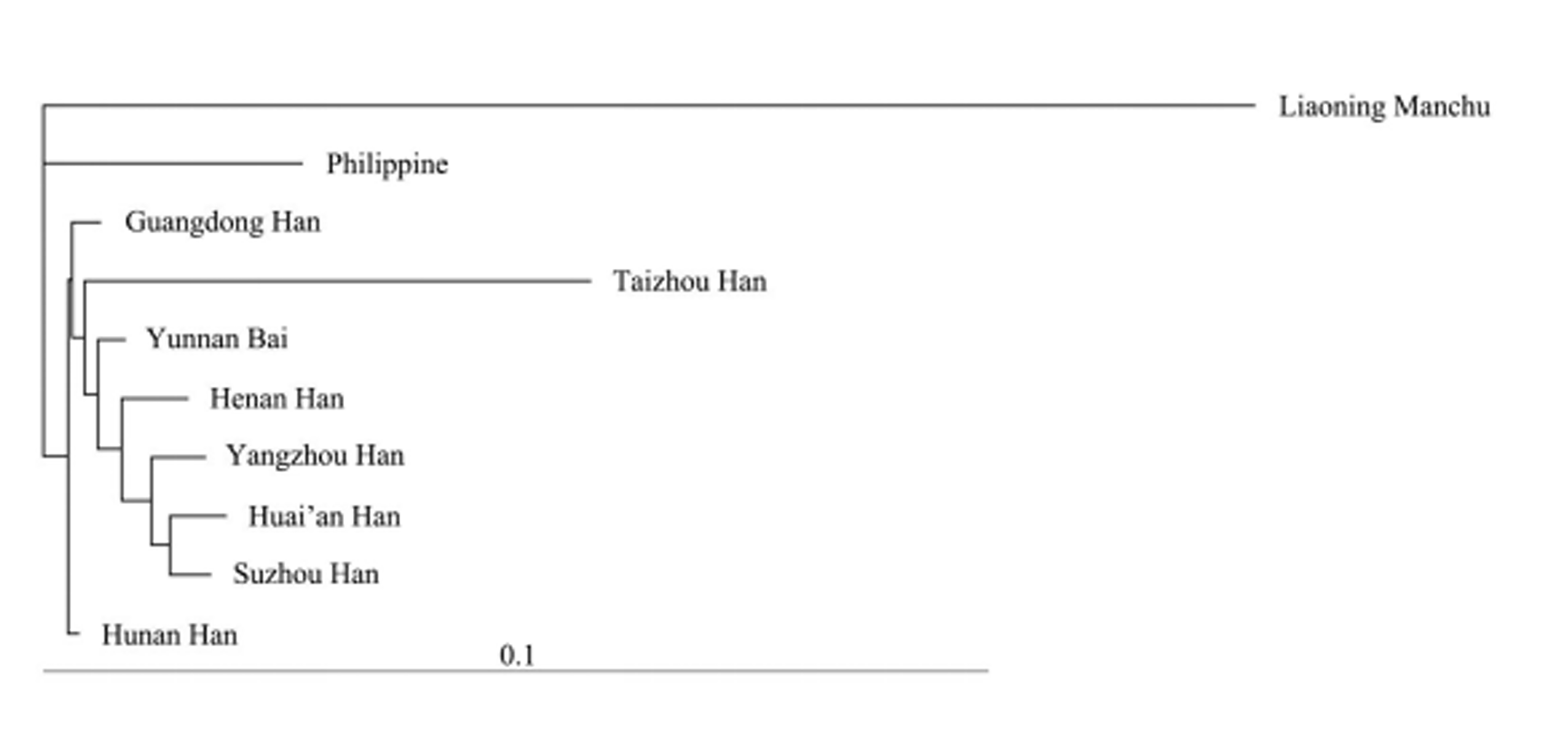

Supplement: 1306430_suppl.zip [file TFSR_A_1306430_SM5902.zip › 1306430_suppl/Figure S2.tif]
